# Modulation of the Functional Components of Growth, Photosynthesis, and Anti-Oxidant Stress Markers in Cadmium Exposed *Brassica juncea* L.

**DOI:** 10.3390/plants8080260

**Published:** 2019-07-31

**Authors:** Dhriti Kapoor, Mahendra P. Singh, Satwinderjeet Kaur, Renu Bhardwaj, Bingsong Zheng, Anket Sharma

**Affiliations:** 1 Department of Botanical & Environmental Sciences, Guru Nanak Dev University, Amritsar 143005, Punjab, India; 2 School of Bioengineering and Biosciences, Lovely Professional University, Delhi-Jalandhar Highway, Phagwara 144411, Punjab, India; 3 State Key Laboratory of Subtropical Silviculture, Zhejiang A&F University, Hangzhou 311300, China

**Keywords:** cadmium toxicity, oxidative stress, antioxidative defense system, photosynthetic pigments

## Abstract

Heavy metals (including Cadmium) are being entered into the environment through various sources and cause toxicity to plants. Response of *Brassica juncea* L. var. RLC-1 was evaluated after exposing them to different concentration of cadmium (Cd) for seven days. Seeds of *B. juncea* were treated with different concentrations of Cd like 0.2–0.6 mM for 7 days, allowing them to grow in Petri-dishes, and seedlings were examined for different physiological responses. Following exposure to Cd, in the seedlings of *B. juncea,* growth parameters (root and shoot length), stress markers (lipid peroxidation and H_2_O_2_ content), secondary metabolites, photosynthetic pigments, and ion analysis, were estimated along with enzymatic and non-enzymatic antioxidants. We observed a significant reduction in root and shoot length after Cd treatment as compared to control seedlings. Malondialdehyde and H_2_O_2_ contents were increased accompanied by enhanced Cd uptake. Activities of antioxidative enzymes were also significantly altered following Cd exposure to the seedlings of *B. juncea*. Conclusively, we suggest that Cd exposure to the seedlings triggered an induction of several defense responses in *B. juncea* including major metabolites.

## 1. Introduction

Plants require optimum level of light, temperature, nutrients, and moisture for their growth and development. Any major deviation from these required optimum conditions affects the plants adversely and may lead to death of the plants. The damage to plants results in retardation of growth, fresh weight, and food production [1,2,3,4,5,6,7,8,9,10]. Heavy metals are one of the major environmental toxicants. Enhancing toxicity of these metals is dangerous for nutritional, biological, and environmental aspects. Chemical nature of metal, its doses, composition, pH of soil, and types of plants are certain factors on which metal toxicity depends [11]. Heavy metal pollution occurs through both natural and anthropogenic sources. Reports suggested that major areas of world have been contaminated by heavy metals through the activities like mining, smelting operations, and agriculture [12,13]. The natural sources like volcanic eruptions, rock outcropping or geologic parent material also contribute their release in the environment. The toxicity of these metals to plants depends upon the oxidation states [14]. The metal toxicity at cellular and molecular level changes various vital activities of plants, which includes deactivation and degradation of enzymes, proteins, displacement/substitution of important metal ions, blocking of functional groups of metabolically important molecules, structural changes, and membrane destabilization [5,14,15,16,17,18]. As a consequence, change in plant metabolism, decrease in respiration, photosynthesis, and change in enzyme activities is observed. In addition, they also stimulate the generation of free oxidants such as hydroxyl radicals (OH), singlet oxygen (^1^O_2_), hydrogen peroxide (H_2_O_2_), and superoxide radicals (O_2_^−^), and thus disturb the redox homeostasis [19,20].

Cadmium (Cd) is present in the soil as free hydrated ions in the form of soil solution or can also be composited with organic or inorganic ligands [21]. Visible effects of high Cd doses in the plants showed reduced growth, leaf chlorosis, closure of stomata, disturbance in water balance, and photosynthetic apparatus of plants [22,23]. According to Al-Yemens [24], reductions in the weights of roots and shoots of *Vigna ambacensis* occur under low Cd concentration. In *Brassica juncea*, application of Cd caused reduction in root and shoot biomass, total chlorophyll content, and total protein content [25]. Increasing concentrations of Cd in soil inhibited germination and seedling ratio of maize seeds [26]. Plants under cadmium stress have decreased net photosynthesis due to inhibition of Rubisco to fix the carbon dioxide [27]. Cd also changes the activities of different metabolic enzymes [28,29,30]. Furthermore, it also binds with sulphydryl groups (SH) of proteins and causes displacement of essential elements, deficiency effects, disruption of structure, and inhibition of enzyme activities [31]. Cd stress stimulates lipid peroxidation, stimulates the synthesis of oxidants like hydrogen peroxide, superoxide, and singlet oxygen and hydrogen radicals, and also alters the activities of antioxidative enzymes [32]. *Brassica juncea*, an important oilseed crop, when cultivated in heavy metals polluted sites, is affected severely towards growth, development, and also the yield. *B. juncea* is mainly grown as a food crop, also used for its medicinal purposes. Mustard green possesses antiseptic, aperitif, diuretic, emetic, and rubefacient properties. It is rich source of antioxidants like flavonoids, indoles, carotenes, lutein, and zea-xanthin [33]. Cadmium toxicity occurs at different concentration and keeping this in mind, we designed an experiment to with different Cd concentrations (0.2, 0.4, and 0.6 mM) to investigate whether it has toxic effect on *B. juncea* plants or it may tolerate lower doses. To assess this, we studied physiological and biochemical parameters using *B. juncea* as plant model system.

## 2. Material and Methods

### 2.1. Plant Material

Certified seeds of *Brassica juncea* L. cv. ‘RLC-1’ used for the current investigation were purchased from Punjab Agriculture University (PAU), Ludhiana, Punjab, India. Seeds of *B*. *juncea* were surface sterilized, followed by treatment with 0, 0.2, 0.4, and 0.6 mM Cd in form of CdCl_2_ solution, and grown on Whatman No. 1 filter paper placed in the autoclaved Petri plates. For each treatment, 3 Petri plates were used with 20 seeds each. Controlled conditions like 25 ± 2 °C, 16 h photoperiod and 175 μmol m^−^^2^ s^−^^1^ light intensity were provided in a seed germinator. Three mL of CdCl_2_ solution was given after every 3 days. Full seedlings were collected on the 7th day for the measurements of morphological parameters and stored at −20 °C for further analysis. Sample collection and further analysis were done using three biological replicates.

### 2.2. Morphological Parameters

Seven-day-old Cd treated or control (untreated) seedlings were harvested and their root length and shoot length were measured.

### 2.3. Oxidative Stress Markers

#### 2.3.1. Malondialdehyde Content

Membrane damage due to exposure to Cd was judged in terms of total malondialdehyde (MDA) content by following the method given by Heath and Packer [34]. Extraction of 1 g seedlings was performed in 0.1% (*w*/*v*) trichloroacetic acid (TCA) and then centrifuged at 5000 rpm. To the supernatant, 20% (*w*/*v*) TCA containing 0.5% (*w*/*v*) TBA was added. Optical density of the supernatant was measured at 532 nm. Extinction coefficient of 155 mM cm^−1^ was taken to measure MDA content.

#### 2.3.2. H_2_O_2_ Content

Hydrogen peroxide (H_2_O_2_) content was estimated by method mentioned by Velikova et al. [35]. Briefly, 500 mg of plant material was taken in which 2 mL of TCA was added and then centrifuged at 12,000 rpm for 15 min. 0.5 mL of 10 mM PBS (phosphate buffered saline) and 1 mL of 1 M potassium iodide was added to 0.5 mL of supernatant. Optical density was taken at 390 nm.

### 2.4. Pigment Analysis

#### 2.4.1. Chlorophyll Content

Level of chlorophyll was measured by using method given by Arnon [36] method. One gram plant tissue was extracted in 80% acetone. The material was then centrifuged at 4 °C for the duration of 20 min at 13,000 rpm. The supernatant was taken to measure chlorophyll content. The optical density was measured at 645 and 663 nm.

#### 2.4.2. Anthocyanin Content

Anthocyanin content was estimated by method described by Mancinelli [37]. Plant tissue (one gram) was crushed in 3 mL of acidified methanol and centrifuged at 4 °C for the time period of 20 min at 13,000 rpm. Then the optical density of supernatant was measured at 530 and 657 nm.

### 2.5. Determination of Sodium and Potassium Ions

Flame emission photometer (Systronics 128) was used to estimate sodium and potassium ion content as mentioned in Chapman [38]. Plant material (0.5 g) was digested in nitric acid (HNO_3_) and perchloric acid (HClO_4_) and total volume was made up to 50 mL by diluting with distilled water. Then the extract was filtered and ion contents were estimated.

### 2.6. Determination of Cd Content and Bioconcentration Factor (BCF)

Atomic Absorption Spectrophotometer (AAS) technique was used to estimate Cd content. Half a gram (0.5 g) dried plant samples were digested in nitric acid and perchloric acid as mentioned in Chapman [38]. Digested samples were filtered and diluted up to 50 mL with distilled water. CdCl_2_ were used as standard solution for the standardization in AAS. Calculation of BCF was done as mentioned by Retamal-Salgado [39].

### 2.7. Estimation of Antioxidant Enzyme Activities

Activities of antioxidative enzymes were determined by the standard methods of Aebi [40] for catalase (CAT) (EC 1.11.1.6), Kono [41] for superoxide dismutase (SOD) (EC 1.15.1.1), Carlberg and Mannervik [42] for glutathione reductase (GR) (EC 1.6.4.2), Dalton et al. [43] for dehydroascorbate reductase (DHAR) (EC 1.8.5.1), and Hossain et al. [44] for monodehydroascorbate reductase (MDHAR) (EC 1.15.1.1).

#### 2.7.1. CAT

The decomposition rate of hydrogen peroxide was followed by reduction in optical density at 240 nm in reaction mixture including 1.5 mL phosphate buffer, 0.7 mL of hydrogen peroxide, and 300 μL of enzyme extract.

#### 2.7.2. SOD

Reaction mixture including 1.73 mL sodium carbonate buffer, 500 μL NBT, and 100 μL Triton X-100 was collected in the test cuvettes. Adding of 100 μL hydroxylamine hydrochloride started the reaction. A total of 70 μL of the plant extract was added after 2 min. Percentage reduction with the rate of NBT decrease was observed as rise in absorbance at 540 nm.

#### 2.7.3. GR

The reaction mixture comprised 50 mM K-phosphate buffer (pH 7.6) (1.45 mL), 3 mM EDTA (0.3 mL), 0.1 mM NADPH (0.3 mL), 1 mM oxidized glutathione (0.3 mL), and 150 μL enzyme extract. The absorbance was read at 340 nm.

#### 2.7.4. DHAR

For execution of this assay, reaction mixture was prepared containing 50 mM phosphate buffer (1.2 mL), 0.1 mM EDTA (0.3 mL), 1.5 mM glutathione (0.3 mL), 0.2 mM dehydroascorbate (0.3 mL), and 400 μL plant extract. A rise in the optical density was estimated at 265 nm.

#### 2.7.5. MDHAR

The assay mixture was consisted of 50 mM of phosphate buffer (1.4 mL), 0.1 mM EDTA (0.3 mL), 0.3 mM NADH (0.3 mL), 0.25 units of ascorbate oxidase, and plant extract (0.5 mL). Decrease in absorbance was measured at 340 nm.

### 2.8. Non-Enzymatic Antioxidants

#### 2.8.1. Ascorbic Acid Content

Level of ascorbic acid was measured by Roe and Kuether [45]. The assay mixture consisted of 100 mg charcoal, 4 mL of double distilled water, 0.5 mL extract of plant, and 0.5 mL of 50% TCA. Then, 0.4 mL of DNPH was taken and the mixture was incubated at 37 °C for 3 h by chilling on ice bath. Sulphuric acid (65% purity, 1.6 mL) was taken and added into it and placed at room temperature for 30 min. Absorbance was taken at 520 nm and 1 mg 100 mL^−1^ ascorbic acid was taken as standard.

#### 2.8.2. Total Phenolic Content

Phenolic content was estimated by Singleton and Rossi [46]. In 0.4 g of dry plant material, 40 mL of 60% ethanol was mixed. Then it was shaken in water at 60 °C for 10 min. Filtered extract was taken and diluted to 100 mL with 60% ethanol. From diluted plant sample, 2.5 mL was taken and re-diluted with 25 mL of distilled water. A two milliliter sample was added with 10 mL of FC reagent and after 5 min, 7.5% of sodium carbonate was added into the reaction mixture. To the mixture, 2 h incubation was given. The optical density was measured at 765 nm. As a reference standard, Gallic acid was taken.

### 2.9. Statistical Analysis

All the experiments were carried out in triplicate and performed three times independently. Data is expressed in mean ± SE. To test the statistical significant difference between the treatments, one way-analysis of variance (ANOVA) was carried out followed by post hoc Tukey’s test using SPSS software. For all the analyses, a *p* value less than 0.05 were considered statistically significant.

## 3. Results

### 3.1. Morphological Parameters

A steep decline in root length was observed with increasing cadmium concentration as compared to control. Approximately three-fold decrease in root length was evident in the seedlings exposed to the highest concentration of cadmium. Slight inhibition in shoot length was found in Cd stressed seedlings. Maximum fall in shoot length was noticed in 0.6 mM Cd stressed seedlings (Table 1).

### 3.2. MDA and H_2_O_2_ Content

**Figure 1:**
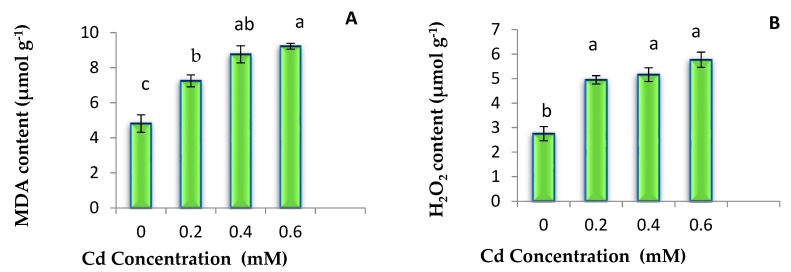
Effects of different Cd treatments on Malondiaeldehyde (MDA) content (**A**) and H_2_O_2_ content (**B**) in 7 days old seedlings of *B. juncea*. Data presented as mean ± SE and means with same letters are not significantly different from each other at *p* < 0.05.

Malondiaeldehyde (MDA) and H_2_O_2_ contents were found lowest in control seedlings. However, with increasing doses of Cd, levels of MDA and H_2_O_2_ were found to increase. Maximum increase in these oxidative stress markers was observed in the seedlings exposed to 0.6 mM Cd (Figure 1).

### 3.3. Pigments

#### 3.3.1. Chlorophyll Content

Cadmium toxicity decreased the total chlorophyll, chl a, and chl b content as compared to control plants. Highest Cd toxicity decreased the level of total chlorophyll 1.99 folds in seven-days old seedlings of *B. juncea* as compared to control. Moreover, a small but significant variation in 0.2 mM and 0.4 mM Cd stressed seedlings was observed for chl a and total chlorophyll. A continuous decline was noticed with increased Cd concentration, where control seedlings possessed highest levels of chl a and chl b (Table 2).

#### 3.3.2. Anthocyanin Content

Application of Cd metal raised the anthocyanin level 2.5 folds from control to 0.6 mM Cd. Moreover, a continuous increase in anthocyanin content was noticed with Cd treatment from 0.2 to 0.6 mM (Table 2).

### 3.4. Cd Uptake and Bioconcentration Factor (BCF) Studies

Results revealed significant accumulation of Cd in *B. juncea* plants (Table 3). It was noticed that Cd accumulation in seedlings was increased with the increasing concentration of Cd. Maximum uptake of Cd was noticed in 0.6 mM treated plants. Control plants were also examined for Cd uptake, but Cd was not detected. Moreover, BCF analysis revealed that Cd uptake efficiency was reduced with increasing Cd treatments (Table 3).

### 3.5. Sodium and Potassium Ion Analysis

Sodium and potassium ions were found to decrease with increasing Cd concentrations (Table 4). Concentration of sodium ion was recorded maximum in control seedlings. Maximum reduction in sodium ions was noticed in the seedlings exposed to highest Cd concentration. Similarly, highest concentration of potassium ions was noticed in the control seedlings. However, significant reduction in potassium content was only noticed in those seedlings which were treated with highest Cd concentration.

### 3.6. Antioxidant Enzyme Activities

Antioxidative defense system got activated after Cd stress and activities of enzymatic antioxidants were noticed to increase. Catalase activity was found minimum in control seedlings, whereas maximum under highest Cd treatment. However, change in CAT activity was not statistically significant among different Cd concentrations. A continuous increase in activity of SOD was noticed due to Cd treatment as compared to control seedlings and 1.54-folds increased SOD activity was observed in 0.6 mM Cd stressed seedlings in comparison to control. Activity of GR enzyme was recorded maximum in 0.4 mM Cd treated seedlings. However, among different Cd treatments, GR activity was not significantly different. Maximum DHAR and MDHAR activities were observed in seedlings grown under maximum Cd concentration. For MDHAR, almost 1.63 folds rise in enzyme activity was noted under highest Cd treatment as compared to control (Table 5).

### 3.7. Level of Non-Enzymatic Antioxidants

A maximum ascorbic acid content was estimated by 1.76 folds in 0.6 mM Cd treated seedlings as compared to control. Moreover, ascorbic acid content was found to be significantly different among all Cd treatments. Similarly, total phenolic content was also increased with Cd treatment and maximum content was found in seedlings grown under highest Cd treatment. However, no significant difference in the phenolic contents of control and 0.2 mM treated seedlings was observed (Table 6).

### 3.8. Correlation Analysis

Correlation analysis between accumulated Cd and other physiological parameters revealed that deposition of Cd in seedlings had a negative impact on growth and stimulated the antioxidative defense system to counterattack oxidative damage and ultimately helps in enhancing the tolerance against Cd (Table 7).

## 4. Discussion

Cadmium limits the crop productivity worldwide as this metal deposits within plant organs and adversely affects the essential physiological processes. Retardation in growth, accumulation of lipid peroxides, alteration in the level of pigments, antioxidants and activities of antioxidative enzymes and generation of ROS were observed due to Cd toxicity in various plant species [47,48,49,50,51,52]. The current study revealed a statistically significant decrease in the root length and shoot length under Cd stress. Due to adverse effects on the roots, nutrition and water supply is also negatively altered, which further affects the growth and physiology of aerial parts of plants. These observations are in agreement with the results of Pandey and Tripathi [53], where Cd treatment to *Albizia procera* seedlings showed retardation of growth in morphological parameters. Cadmium stress adversely affected root length, shoot length, leaf area, and biomass of *A. procera*. Similarly, in another study supplement of 10 ppm dose of Cd^2+^ and Cr^6+^ to *Medicago sativa* plants drastically inhibited growth as compared to the control plants. Further, when their doses were raised to 20 ppm, growth was diminished by 62.0% and 65.0%, respectively [54]. Inhibitory effect of Cd was more evident in the growth of roots as compared to others as roots are the first position of exposure to the ions of Cd through apoplastic transport that leads to maximum accumulation [55,56] The accumulation of Cd suppresses elongation of root and also responsible for deformation of root due to direct inhibition of root and shoot metabolism [57]. Correlation analysis of accumulated Cd in seedlings with other physiological parameters also confirmed the negative impact of Cd upon those parameters (Table 7).

Chlorophyll rapidly declined with increasing the doses of Cd in the present study. Reduction of Fe due to Cd metal causes chlorosis in leaves and also negatively affects chlorophyll metabolism. Heavy metals cause degradation of the micronutrients required for the plants and thus lead to inhibition of the level of pigments like chlorophyll [58]; this has been found as one of major reasons for the impairment of photosynthesis and inhibition of growth in plants under the effect by this metal. In the current study, higher Cd concentration showed negative impact on the sodium and potassium contents. Photosynthesis is the basic phenomena of the plant which regulates the development and growth of plants [59], and previous reports have documented that Cd can significantly degrade the chlorophylls and inhibit the photosynthesis [60]. Similarly, our results also showed statistically significant down-regulated chlorophyll levels thereby depicting degradation, or inhibition of their biosynthesis following Cd exposure even at the lowest the concentration used (0.2 mM).

Other important components that are present in the plants are anthocyanins, these are bioactive secondary metabolites of the plants wherein biological functions act as powerful antioxidant machinery and can also lead to the chelation of metal ions [61]. Stimuli due to unsuitable environment like exposure to Cd are well documented to stimulate anthocyanins accumulation in the plants [62]. Interestingly, in this study, Cd exposure had statistically significant higher anthocyanins in *B. Juncea* even at 0.2 mM Cd treated groups.

The treatment of Cd metal to *B. juncea* plants in the present study revealed that metal stress enhanced the level of antioxidants and activities of antioxidative enzymes. There was a significant increase in the ascorbic acid, total phenolic content, and activities of CAT, SOD, GR, DHAR, and MDHAR in Cd stressed *B. juncea* plants. SOD has a significant contribution in the protection against reactive oxygen species, produced as by-products of biological oxidations. These observations have also been documented by Zhang et al. [63] and Nehnevajova et al. [64]. A previous report by Najeeb et al. [65] evidenced a Cd modulated antioxidant defense system of the seedlings of *B. juncea*. The exposure of Cd inhibited the activities of antioxidant markers like SOD, POD, GST, and PPO and also significantly stimulated the activities of CAT, APOX, GPOX, DHAR, and GR. The stress caused by Cd inhibits enzymatic markers may be due to impairment of enzyme synthesis or due to inhibition of growth along with accumulation of H_2_O_2_ [65]. In this context, superoxide dismutase (Cu-Zn SOD) acts as a tier of cellular defense machinery that converts superoxide anion to H_2_O_2_. Then, these H_2_O_2_ is converted to water molecule majorly through antioxidant enzymes like catalases and peroxidases. Interestingly, ascorbate peroxidase reduces hydrogen peroxide through ascorbic acid-glutathione pathway that uses antioxidant enzymatic markers [66]. The glutathione peroxidases and peroxiredoxins also eliminate H_2_O_2_ and other organic hydroperoxides using non-enzymatic antioxidant markers like gluathione, thioredoxin, and glutaredoxin as nucleophiles [67]. The synthesis of phenolic compounds acts efficiently on metal decontamination as antioxidants [68]. The exposure to Cd reduces intracellular GSH contents and Cd exposure has also been reported to reduce GSH content in a number of plant species [69,70]. Nazar and his colleagues [71] explained that the reduction of GSH levels may be due to stress imposed by Cd, as it is known to prompt the formation of phytochelatins (oligomers of reduced glutathione) through increasing phytochelatin synthase activity, and these phytochelatins can conjugate with free ions of Cd and get sequestered into the vacuoles.

The present investigation revealed the increased level of MDA, H_2_O_2_ and also enhanced production of free radicals and oxidative damage due to membrane destabilization in plants being exposed to metal. MDA is a cytotoxic by product of lipid peroxidation and also acts as a marker of generation of free radical [63]. High doses of heavy metals lead to the production of ROS like H_2_O_2_, and it is the primary response of plants under stress. ROS generation occurs in the presence of oxidative stress or directly through Haber–Weiss reactions [72] and this supports the present study as we have found similar results in this context.

## 5. Conclusions

From the present study it is concluded that Cd toxicity adversely affects the seedlings even at low concentration as reported earlier (0.2 mM) in terms of increased production of ROS, higher lipid peroxidation, and by negatively affecting the photosynthetic pigment system. While in response to the detrimental effects of metal, defense responses of *B. juncea* seedlings got activated by the action of metabolites like phenols and compatible solutes. Besides, antioxidative defense system of seedlings helped in scavenging of free radicals generated during metal stress. It was also noticed that the ROS content was initially increased with Cd treatment, but no significant change was noticed between middle and highest Cd treatment. This might be due to the enhanced ROS scavenging as a result of activated antioxidative system. Furthermore, growth of seedlings was reduced with Cd treatment but there was also no statistical difference in growth parameters among different Cd treatments. These facts revealed that activated antioxidant system resulted in providing better Cd tolerance to seedlings.

## Figures and Tables

**Table 1:** Effect of Cadmium (Cd) metal on growth parameters of 7 days old seedlings of *B. juncea*.

Cd Conc. (mM)	Root Length (cm)	Shoot Length (cm)
0.0	11.7 ± 1.57 ^a^	4.37 ± 0.22 ^a^
0.2	7.63 ± 0.41 ^b^	4.17 ± 0.2 ^ab^
0.4	5.97 ± 0.12 ^b^	3.43 ± 0.2 ^ab^
0.6	3.93 ± 0.5 ^b^	3.33 ± 0.26 ^b^

Data represents mean ± SE, and the experiment was repeated three times independently. Means with same letters are not significantly different from each other at *p* < 0.05.

**Table 2:** Effect of Cd metal on pigment contents of 7 days old *B. juncea* seedling.

Cd Conc. (mM)	Total Chl (mg g^−1^ FW)	Chl a (mg g^−1^ FW)	Chl b (mg g^−1^ FW)	Anthocyanin (mg g^−1^ FW)
0.0	26.75 ± 0.77 ^a^	16.78 ± 0.56 ^a^	7.54 ± 0.47 ^a^	2.41 ± 0.28 ^b^
0.2	21.69 ± 0.9 ^b^	15.04 ± 0.93 ^a^	5.33 ± 1.29 ^ab^	3.6 ± 0.32 ^b^
0.4	18.56 ± 0.39 ^c^	11.59 ± 0.44 ^b^	6.12 ± 0.41 ^a^	5.09 ± 0.41 ^a^
0.6	13.38 ± 0.39 ^d^	7.1 ± 0.23 ^c^	2.24 ± 0.19 ^b^	6.02 ± 0.11 ^a^

Data represents mean ± SE, *n* = 3 and experiment was repeated three times independently. Means with same letters are not significantly different from each other at *p* < 0.05.

**Table 3:** Data showing Cd accumulation and bioconcentration factor in 7 days old seedlings of *B. juncea* grown under different Cd treatments.

Cd Conc. (mM)	Cd Accumulation in Seedlings (μg g^−1^ DW)	BCF
0.2	69.13 ± 5.14 ^b^	1.85
0.4	83.39 ± 2.35 ^ab^	1.13
0.6	89.68 ± 2.87 ^a^	0.82

Data represents mean ± SE, *n* = 3, and the experiment was repeated three times independently. Means with same letters are not significantly different from each other at *p* < 0.05.

**Table 4:** Effect of Cd on contents of sodium and potassium ions in 7 days old seedlings of *B. juncea*.

Cd Conc. (mM)	Sodium Ion (ppm)	Potassium Ion (ppm)
0.0	6.38 ± 0.57 ^a^	5.82 ± 0.59 ^a^
0.2	5.89 ± 0.59 ^a^	5.25 ± 0.29 ^ab^
0.4	4.99 ± 0.49 ^b^	5.5 ± 0.62 ^ab^
0.6	4.42 ± 0.36 ^b^	4.24 ± 0.79 ^b^

Data represents mean ± SE, *n* = 3, and the experiment was repeated three times independently. Means with same letters are not significantly different from each other at *p* < 0.05.

**Table 5:** Effect of Cd metal on activities of enzymatic antioxidants in 7 days old seedlings of *B. juncea*.

Cd Conc. (mM)	CAT (UA mg^−1^ Protein)	SOD (UA mg^−1^ Protein)	GR (UA mg^−1^ Protein)	DHAR (UA mg^−1^ Protein)	MDHAR (UA mg^−1^ Protein)
0.0	6.43 ± 0.19 ^b^	5.43 ± 0.35 ^b^	7.63 ± 0.27 ^b^	8.33 ± 0.49 ^c^	11.29 ± 0.33 ^c^
0.2	8.27 ± 0.35 ^a^	6.38 ± 0.21 ^b^	9.66 ± 0.40 ^a^	10.25 ± 0.33 ^b^	14.65 ± 0.44 ^b^
0.4	8.51 ± 0.53 ^a^	7.75 ± 0.25 ^a^	10.87 ± 0.54 ^a^	11.25 ± 0.26 ^ab^	16.94 ± 0.62 ^a^
0.6	8.94 ± 0.32 ^a^	8.39 ± 0.24 ^a^	9.93 ± 0.47 ^a^	12.21 ± 0.29 ^a^	18.46 ± 0.55 ^a^

Data represents Mean ± SE, *n* = 3, and the experiment was repeated three times independently. Means with same letters are not significantly different from each other at *p* <0.05.

**Table 6:** Effect of Cd metal on non-enzymatic antioxidants in 7 days old seedlings of *B. juncea*.

Cd Conc. (mM)	Ascorbic Acid (mg g^−1^ FW)	Total Phenolic Content (mg g^−1^ FW)
0.0	8.63 ± 0.25 ^d^	6.86 ± 0.20 ^c^
0.2	10.34 ± 0.49 ^c^	7.89 ± 0.49b ^c^
0.4	12.97 ± 0.31 ^b^	8.89 ± 0.35 ^ab^
0.6	15.23 ± 0.41 ^a^	9.96 ± 0.15 ^a^

Data represents mean ± SE, *n* = 3, and the experiment was repeated three times independently. Means with same letters are not significantly different from each other at *p* < 0.05.

**Table 7:** Data showing correlation between Cd accumulation and different parameters.

Parameter	Correlation Coefficient	*p* Value
Root length	−0.8738	0.0002
Shoot length	−0.6936	0.0123
MDA content	0.9189	2.39 × 10^−5^
H_2_O_2_ content	0.9347	8.38 × 10^−6^
Total chlorophyll content	−0.8791	0.0001
Anthocyanin content	0.8566	0.0003
Sodium ion content	−0.6271	0.0291
Potassium ion content	−0.3413	0.2775
CAT activity	0.8703	0.0002
SOD activity	0.8365	0.0006
GR activity	0.8729	0.0002
DHAR activity	0.8856	0.0001
MDHAR activity	0.9227	1.89 × 10^−5^
Ascorbic acid content	0.8186	0.0011
Total phenolic content	0.8241	0.0009

## Abbreviations

H_2_O_2_	hydrogen peroxide
CAT	catalase
SOD	superoxide dismutase
GR	glutathione reductase
DHAR	dehydroascorbate reductase
MDHAR	monodehydroascorbate reductase
